# Innovative Cancer Immunotherapy with MAGE-A3 mRNA Cancer Vaccines

**DOI:** 10.3390/cancers16193428

**Published:** 2024-10-09

**Authors:** Kangchan Choi, Hyorim Jeong, Do Hyun Lee, Ji Won Lee, Ju-Eun Hong, Jin Ee Baek, Yong Serk Park

**Affiliations:** 1 School of Medicine, Trinity Medical Sciences University, Roswell, GA 30075, USA; 2 College of Medicine and Engineering, University of Illinois Chicago, Chicago, IL 60612, USA; 3 Department of Biomedical Laboratory Science, Yonsei University, Wonju 26493, Republic of Korea; 4 Department of Pathologic Laboratory Research, Institute of Occupation and Environment, Incheon 21417, Republic of Korea

**Keywords:** mRNA cancer vaccine, cancer immunotherapy, tumor-associated antigen, melanoma-associated antigen A3

## Abstract

**Simple Summary:**

In this study, we developed an mRNA cancer vaccine by encapsulating melanoma-associated antigen A3 (MAGE-A3) mRNA in lipid nanoparticles made of O,O′-dimyristyl-N-lysyl aspartate (DMKD) and phosphatidylserine (PS). These nanoparticles demonstrated stability, preserving mRNA integrity even after extended refrigeration. Immunization with this vaccine significantly inhibited tumor growth in mice and activated strong immune responses. This suggests that the MAGE-A3 mRNA vaccine using DMKD-PS nanoparticles could provide both therapeutic and preventive benefits for tumors associated with this antigen.

**Abstract:**

Cancer causes over 10 million deaths annually worldwide and remains a significant global health challenge. This study investigated advanced immunotherapy strategies, focusing on mRNA vaccines that target tumor-specific antigens to activate the immune system. We developed a novel mRNA vaccine using O,O′-dimyristyl-N-lysyl aspartate (DMKD) to improve stability and phosphatidylserine (PS) to enhance antigen presentation to immune cells. This vaccine, containing melanoma-associated antigen A3 (MAGE-A3) mRNA encapsulated within lipid nanoparticles (LNPs), was evaluated for its therapeutic potential against colorectal cancer. Our findings demonstrated that MAGE-A3 mRNA-containing DMKD-PS LNPs significantly reduced tumor size and weight, effectively combating metastatic cancer. The vaccine elicited a robust immune response, increasing specific immunoglobulin and cytokine levels without causing histotoxicity in major organs. These results confirm that the DMKD-PS-based MAGE-A3 mRNA vaccine holds promise for cancer prevention and treatment.

## 1. Introduction

Cancer claims over 10 million lives annually and is the second leading cause of mortality worldwide [1]. The primary treatment methods include surgery, radiotherapy, and chemotherapy [2]. While surgical removal of tumors is often essential, it carries significant risks [3]. In some cases, surgery may unintentionally cause cancer metastasis or stimulate the accelerated growth of cancer cells [4]. Radiotherapy and chemotherapy, which target tumors in surgically inaccessible areas, can also have severe side effects, as they damage both healthy and cancerous cells, leading to issues such as hair loss, vomiting, and dysfunction of the heart or liver [5].

To address these limitations, a variety of anticancer therapies have been developed, with immunotherapy emerging as a promising option [6]. This approach harnesses the patient’s immune system to eliminate tumors, including immune cell therapy, immune checkpoint inhibitors, and cancer vaccines [7]. Cancer vaccines aim to stimulate the immune system to recognize and attack tumor antigens, helping the body eliminate malignant cells [8]. These vaccines typically involve the administration of tumor antigens, paired with adjuvants, to trigger an immune response in cells like antigen-presenting cells (APCs) [9]. Messenger RNA (mRNA) from autologous tumors can stimulate a specific cytotoxic T lymphocyte (CTL) response, effectively promoting the elimination of cancer cells [10].

Using total RNA as a cancer vaccine can induce immune responses against a wide range of tumor antigens, thereby reducing the risk of tumor escape. Pathogens entering the body are recognized by unique molecular markers, which activate an immune response. T cells identify tumor cells through tumor-associated antigens (TAAs), which are overexpressed in cancer cells. These antigens are pivotal to cancer immunotherapy, as they help the immune system selectively target and attack cancer cells. Focusing on TAAs is crucial for developing effective cancer vaccines that boost the body’s natural defense mechanisms [11].

Melanoma-associated antigen A3 (MAGE-A3) is a common TAA found in melanoma [12]. MAGE-A3 is frequently expressed in melanoma and other cancers, including pleomorphic sarcoma and gastric cancer [13]. T cells recognize MAGE-A3, which enhances its potential as a target for cancer vaccines. Since MAGE-A3 is minimally expressed in normal tissues, and only during specific developmental stages, it typically does not trigger autoimmune reactions.

In previous studies, we demonstrated that lipid nanoparticles (LNPs) based on O,O′-dimyristyl-N-lysyl aspartate (DMKD) could stably encapsulate mRNA molecules, preserving their integrity for months. We successfully developed a MAGE-A3 mRNA cancer vaccine specifically targeting tumors expressing the MAGE-A3 antigen. This study highlights the vaccine‘s potential preventive and therapeutic effects against MAGE-A3-associated tumors, representing a significant advancement in cancer treatment strategies.

## 2. Results and Discussion

### 2.1. Synthesis of Melanoma-Associated Antigen A3 mRNA

**Figure 1:**
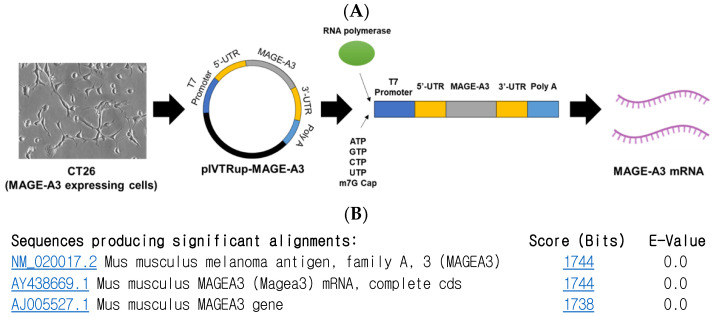
Melanoma-associated antigen A3 (MAGE-A3) mRNA synthesis. (**A**) Utilizing the IVT method for synthesizing GFP mRNA, the entire MAGE-A3 coding sequence was incorporated into the plasmid via subcloning. (**B**) Following sequencing and gene alignment, successful insertion of MAGE-A3 was confirmed.

To synthesize MAGE-A3 mRNA using the in vitro transcription (IVT) method, a pIVTRUP-MAGE-A3 plasmid was constructed (Figure 1A). The MAGE-A3 gene sequence, derived from CT26 cells (a mouse colon cancer cell line), was inserted into the plasmid. Subsequent sequencing and gene alignment confirmed the successful insertion of the MAGE-A3 gene into the prepared pIVTRUP-MAGE-A3 plasmid (Figure 1B).

### 2.2. In Vitro Transfection of MAGE-A3 mRNA

Previously, we demonstrated that a novel formulation of O,O′-dimyristyl-N-lysyl aspartate–phosphatidylserine (DMKD-PS) lipid nanoparticles (LNPs) could deliver green fluorescent protein (GFP) mRNA to immune cells such as macrophages and colorectal cancer cells [14]. In the present study, we employed the same formula to assess the anticancer immunotherapeutic efficacy of MAGE-A3 mRNA. MAGE-A3 mRNA was formulated with various LNPs, including DMKD, DMKD-PS, ALC-0315, and SM-102, and transfected into mouse macrophages.

**Figure 2:**
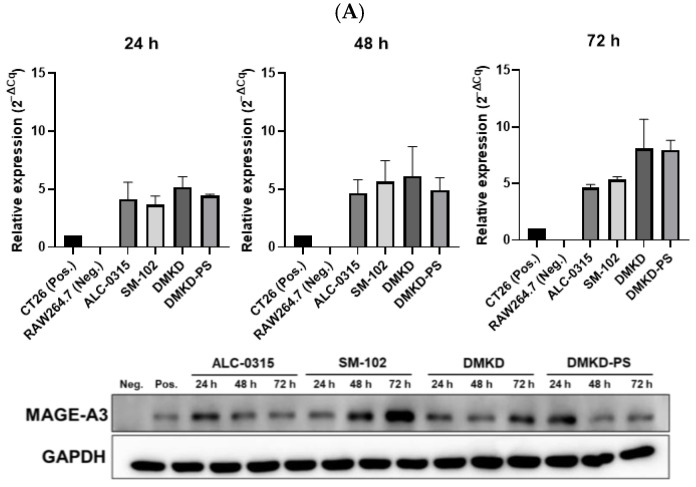

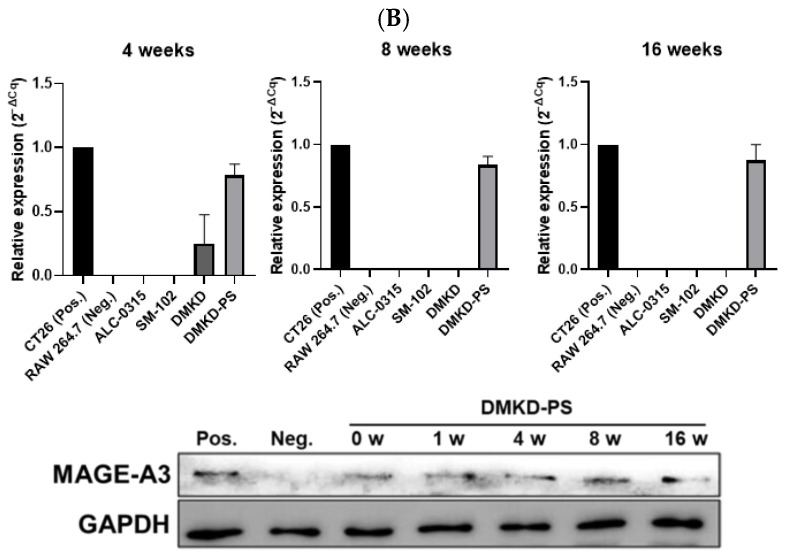
Relative expression of MAGE-A3 after in vitro mRNA transfection. (**A**) RNA and protein expression following transfection with various prepared LNPs and MAGE-A3 mRNA measured for 72 h. (**B**) Stability of varied LNPs/MAGE-A3 mRNA complexes refrigerated for 16 weeks was evaluated in terms of RNA transcription and protein production.

To confirm the successful transfection of MAGE-A3 mRNA, we analyzed the levels of MAGE-A3 mRNA in transfected RAW 264.7 cells using quantitative reverse transcription PCR (RT-qPCR) and Western blotting. At 24, 48, and 72 h post-transfection, all tested LNPs effectively delivered MAGE-A3 mRNA into RAW 264.7 cells, with RNA expression levels generally increasing over time. Notably, at 72 h, DMKD and DMKD-PS LNPs exhibited higher relative RNA expression levels than ALC-0315 and SM-102 LNPs. Western blot analysis further confirmed the successful translation of the MAGE-A3 protein after transfection (Figure 2A).

The stability of MAGE-A3 mRNA-encapsulating LNPs was also evaluated after 16 weeks of refrigeration. After four weeks, ALC-0315 and SM-102 LNPs lost the ability to deliver MAGE-A3 mRNA into RAW 264.7 cells. DMKD LNPs showed a decline in mRNA delivery efficiency after eight weeks of storage. In contrast, DMKD-PS LNPs maintained their structural integrity and successfully delivered mRNA, yielding efficient intracellular RNA transfection even after 16 weeks of refrigeration. Remarkably, RAW 264.7 cells transfected with DMKD-PS LNPs continued to express the MAGE-A3 antigen after 16 weeks (Figure 2B). Thus, DMKD-PS LNPs demonstrated superior mRNA delivery capabilities for antitumor immunization compared to other formulations, including FDA-approved LNPs.

### 2.3. Prevention of Tumorigenesis by Anticancer Vaccines Containing MAGE-A3 mRNA

**Figure 3:**
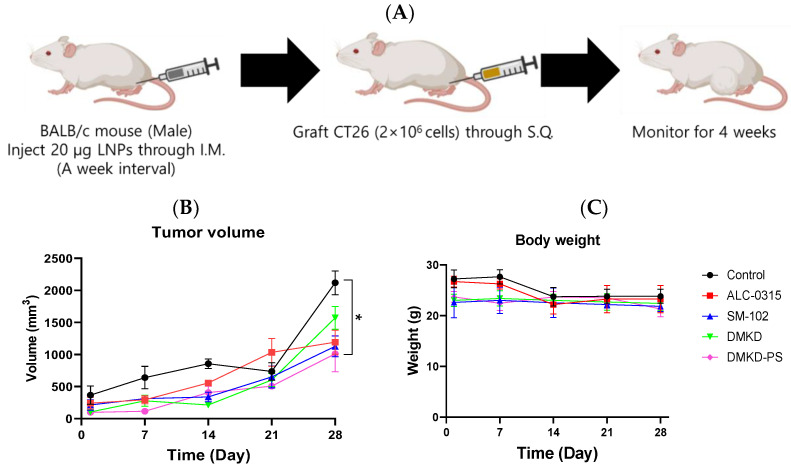
Preventive effects of MAGE-A3 mRNA-containing LNPs on tumor growth. (**A**) Experimental design of immunization and challenge of CT26 tumor cells in mice. The changes in (**B**) tumor size and (**C**) body weight were monitored for 28 d. * *p* < 0.05.

To evaluate whether the MAGE-A3 mRNA anticancer vaccine could suppress tumor development, CT26 tumor cells were implanted into male BALB/c mice that had been immunized with the mRNA cancer vaccines two weeks prior (Figure 3A). Tumor size was monitored for four weeks, and no additional drugs were administered besides the mRNA anticancer vaccine formulations.

Despite vaccination, CT26 tumors formed in all immunized mice. However, the tumor growth rate was generally slower in vaccinated mice compared to the unvaccinated control group. Notably, the tumor volume increased rapidly after the third week of observation. By Week 4, tumors in DMKD-PS mRNA-vaccinated mice were approximately 1.1 times larger than those in Week 3, while untreated control mice exhibited over a threefold increase in tumor size (Figure 3B). In contrast, tumor sizes in mice treated with ALC-0315, SM-102, or DMKD alone increased by an average of 1.5- to 3-fold. Body weight measurements showed a slight decline at four weeks, indicating that none of the mRNA anticancer vaccines caused significant toxicity leading to severe weight loss (Figure 3C,D).

### 2.4. Histopathological Assessment after DMKD-PS MAGE-A3 mRNA Anticancer Vaccination

**Figure 4:**
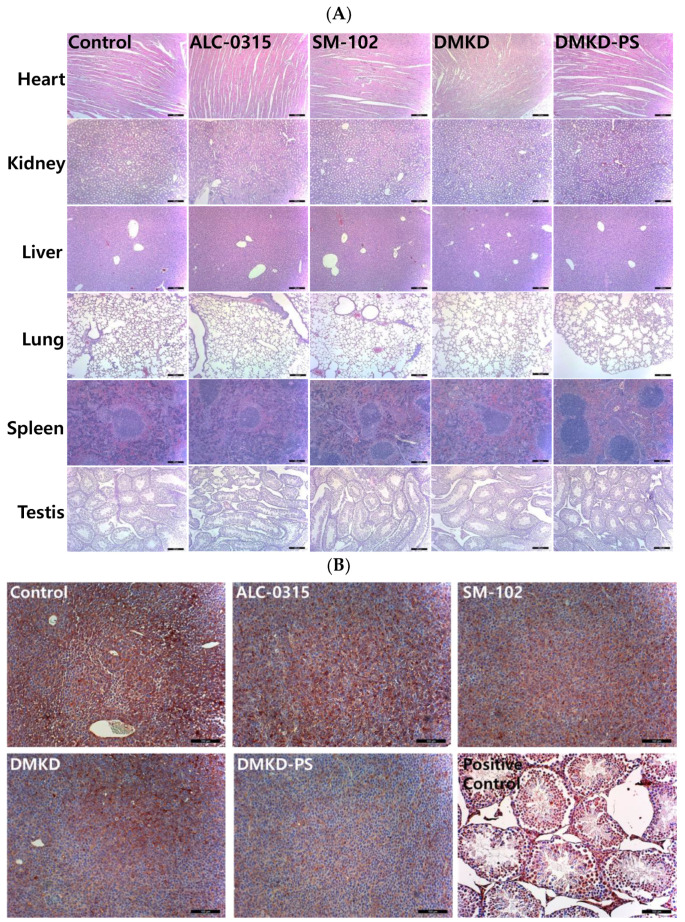
Histopathological analysis of major internal organs and tumors in mice treated with the mRNA anticancer vaccine. (**A**) On the 28th day post-inoculation, major organs were harvested from tumor-bearing mice and processed for histopathological examination. Magnification: ×100 (scale bar = 100 μm). (**B**) Tumor tissues extracted from vaccinated mice were analyzed immunohistochemically using an antibody specific to MAGE-A3. Magnification: ×200 (scale bar = 100 μm).

Histological evaluations were conducted using hematoxylin and eosin (H&E) staining to assess potential systemic histotoxicity induced by the mRNA-based anticancer vaccines. Major organs, including the heart, kidneys, spleen, liver, and lungs, were excised from the vaccinated mice and subjected to microscopic examination following H&E staining (Figure 4A). The histological analysis revealed no abnormalities, such as inflammation or necrosis, in any of the organs.

Additionally, to investigate potential autoimmune responses to MAGE-A3 expression, testicular tissues, which naturally express the MAGE-A3 antigen, were also examined microscopically (Figure 4B). The examination showed no abnormal tissue morphology in the testicular tissues of mice vaccinated with MAGE-A3 mRNA using DMKD-PS LNPs. Therefore, the mRNA-complexed DMKD-PS LNPs did not induce any abnormal toxicity in the major organs or testes.

### 2.5. Survival Rate of Tumor-Metastasized Mice Vaccinated with MAGE-A3 mRNA

**Figure 5:**
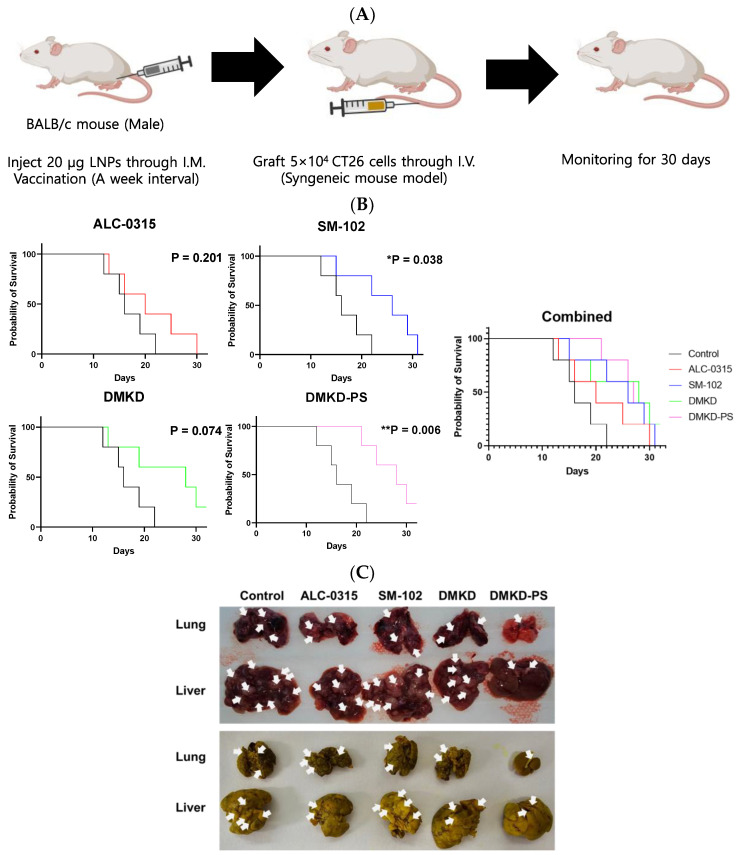
Survival of mice immunized with MAGE-A3 mRNA and then challenged with tumor cells. (**A**) Schematic image of immunized mice were intravenously injected with CT26 cells to artificially induce a tumor metastasis model. (**B**) Survival rate of the mice vaccinated with MAGE-A3 mRNA in various LNPs were intravenously injected with CT26 cells to induce tumor metastasis artificially. (**C**) Entire livers and lungs with tumors were observed before (**upper**) and after (**bottom**) fixation in Bouin’s solution with picric acid. Tumors were identified and marked with white arrows. Tumors typically presented as dark red or white nodules and appeared as bright-yellow nodules upon fixation with Bouin’s solution. * *p* < 0.05, ** *p* < 0.01.

To evaluate the effect of the MAGE-A3 mRNA anticancer vaccine on inhibiting tumor metastasis, CT26 cells were administered via the tail vein of mice that had been immunized with the mRNA formulated in various LNPs (Figure 5A). Mouse survival was monitored for 30 days following CT26 cell injection. Most mice treated with ALC-0315, SM-102, or DMKD LNPs likely succumbed to metastatic tumors within the 30-day observation period. In contrast, mice administered MAGE-A3 mRNA in DMKD-PS LNPs exhibited significantly longer survival, with a stronger *p*-value (0.006) compared to the untreated control group. Notably, all mice inoculated with DMKD-PS LNPs survived until Day 20, indicating a prolonged survival period relative to other groups (Figure 5B).

Postmortem examinations were conducted on the last deceased or surviving mouse from each group. The lungs and liver were excised to assess the extent of tumor metastasis. Comparative analysis of metastasized tumor nodules in the lungs and liver revealed that mice treated with DMKD-PS LNPs had the least metastasis compared to those treated with other LNPs (Figure 5C). The prolonged survival and reduced metastasis observed in the DMKD-PS LNP-treated mice suggest that the DMKD-PS LNP system is a more effective mRNA delivery vehicle for cancer therapy in vivo.

### 2.6. Cancer Treatment with MAGE-A3 mRNA-Containing Anticancer Vaccines

**Figure 6:**
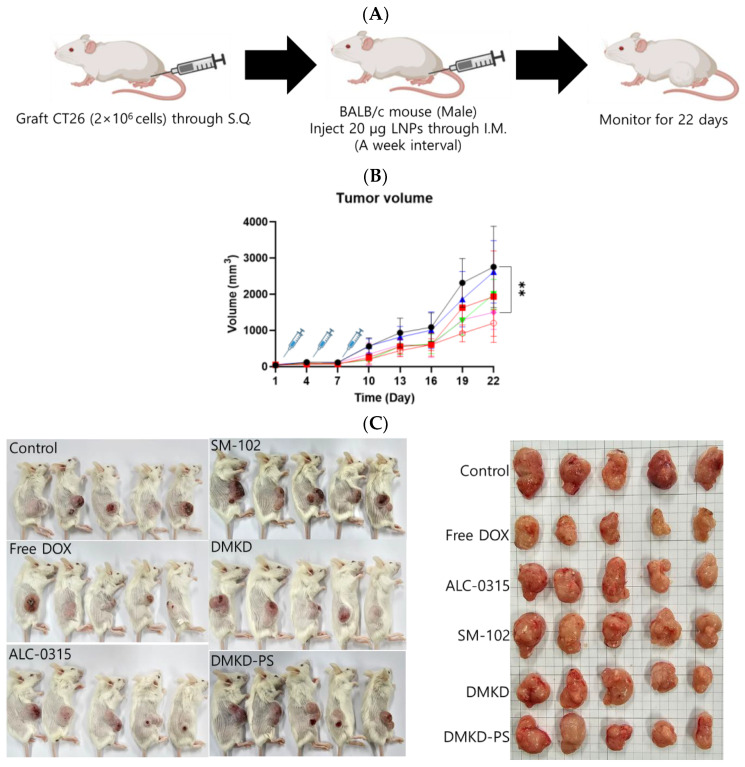

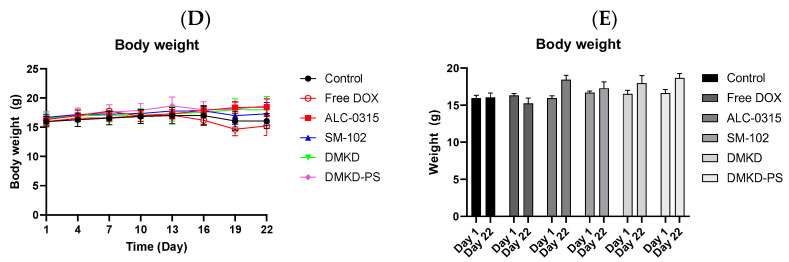
The in vivo anticancer treatment efficacy of MAGE-A3 mRNA-containing LNPs. (**A**) A schematic illustration providing an overview of the experimental design. (**B**) Tumor growth was monitored twice a week after trice injection of the mRNA vaccines. (**C**) At the endpoint of the experiment, photographs of the sacrificed tumor-bearing mice and the excised tumors were captured, visually documenting the treatment outcomes. (**D**,**E**) The body weight of the mice was measured throughout the experimental period. ** *p* < 0.01.

Mice syngeneically transplanted with CT26 cells were used to evaluate the therapeutic potential of MAGE-A3 mRNA cancer vaccines (Figure 6A). MAGE-A3 mRNA, encapsulated in various LNP formulations, was intramuscularly administered to tumor-bearing mice. A group of tumor-bearing mice was also treated with the well-established anticancer drug doxorubicin, allowing for a comparison of the therapeutic efficacy of the MAGE-A3 mRNA vaccines. Tumor growth observation was terminated on Day 22, as the tumor size in untreated control mice exceeded 10% of their body weight.

Unexpectedly, the SM-102 formulation was the least effective in inhibiting tumor growth (Figure 6B,C). Nevertheless, tumor growth suppression or a reduction in tumor volume was observed in the other treatment groups. The DMKD-PS formulation demonstrated antitumor efficacy comparable to that of doxorubicin administered subcutaneously via the tail vein. However, mice treated with doxorubicin experienced weight loss during the monitoring period (Figure 6D), while those treated with mRNA LNP vaccines gained weight by the study‘s endpoint (Figure 6E). Although some weight gain may be partly due to tumor growth, the untreated control group showed minimal changes in body weight. These results suggest that the MAGE-A3 mRNA anticancer vaccine formulated in LNPs did not induce significant toxicity in vivo.

### 2.7. Histopathological Assessment after DMKD-PS mRNA Vaccination

**Figure 7:**
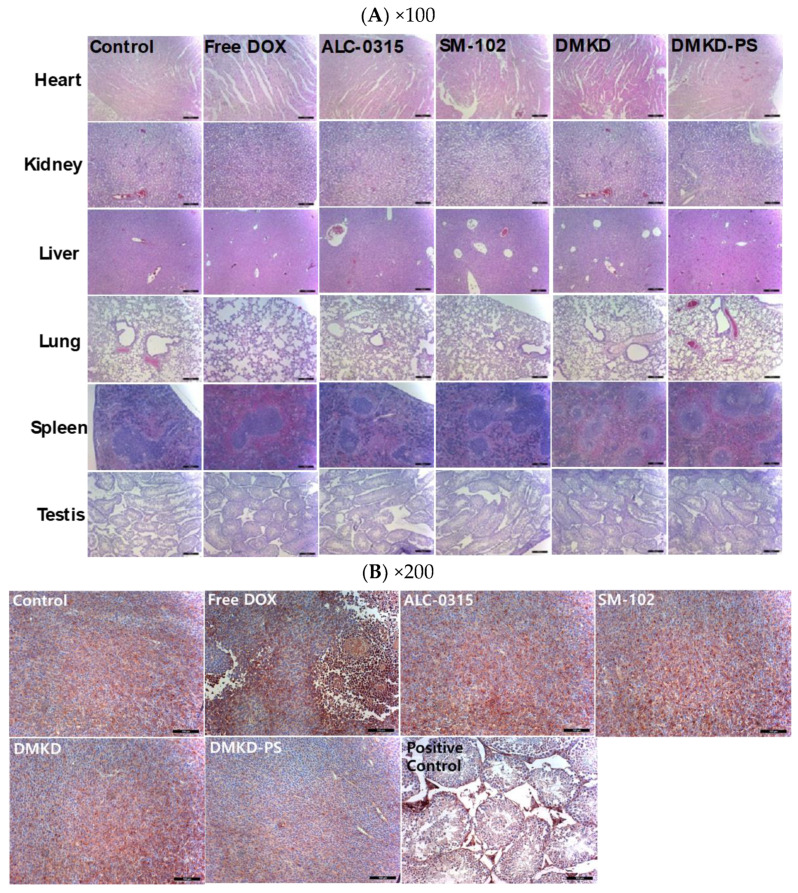
Histopathological examination of major internal organs and tumors of mice injected with MAGE-A3 mRNA vaccine. (**A**) Twenty-two days after the first treatment with MAGE-A3 mRNA vaccines, major organs were collected from the tumor-bearing mice. The organs were paraffin-embedded, sectioned, and subjected to H&E staining for histopathological analysis. Magnification: ×100 (scale bar = 100 μm). (**B**) Tumor tissues were immunohistochemically stained using an anti-MAGE-A3 antibody and examined under a light microscope for detailed analysis. Magnification: ×200 (scale bar = 100 μm).

Following the intramuscular administration of MAGE-A3 mRNA vaccines for cancer treatment or tumor growth inhibition, major organs, including the liver, lungs, heart, kidneys, and spleen, were stained with H&E for histological examination (Figure 7A). Additionally, tumor cross-sections were analyzed using immunohistochemistry (IHC) staining with anti-MAGE-A3 antibodies, focusing on deeper tumor layers (Figure 7B). Mild myocardial fissures were noted in the heart tissues of mice treated with cancer vaccines, but these fissures were absent in the control and DMKD-PS-treated groups. No other significant histopathological abnormalities were detected in any of the tissues. Similarly, no adverse effects were observed in the testicles, confirming that the MAGE-A3 cancer vaccines did not impact the naturally expressed MAGE-A3 in this organ.

Interestingly, IHC analysis of tumor cross-sections revealed the lowest expression of MAGE-A3 in most tumor tissues from mice treated with the DMKD-PS-based cancer vaccine. This suggests potential interactions between tumor cells and MAGE-A3-expressing immune cells and/or anti-MAGE-A3 antibodies. In contrast, treatment with doxorubicin significantly disrupted the internal structure of the tumor tissue, showing necrotic changes that hindered tumor growth, consistent with the tumor treatment process.

### 2.8. Verification of Anti-Tumoral Immunity Induced by MAGE-A3 mRNA Vaccination

**Figure 8:**
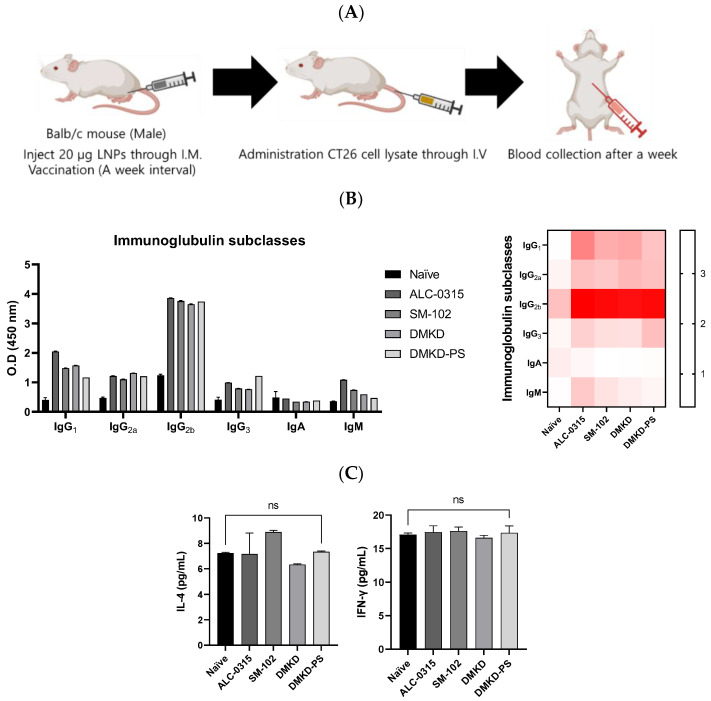
Immunoglobulin subtyping and cytokine profiling following MAGE-A3 mRNA anticancer vaccination. (**A**) A schematic representation of blood collection after the injection of CT26 cell lysate into the tail vein of an immunized mouse. (**B**) Immunoglobulin subtypes and (**C**) cytokines, specifically IL-4 and IFN-γ, were quantified a week later.

Various formulations were administered to mice to elucidate the antitumor immune responses elicited by MAGE-A3 mRNA vaccines, followed by a challenge with CT26 cell lysates (Figure 8A). One week later, significant changes in the components of humoral immunity, such as immunoglobulins and the cytokine Interleukin-4 (IL-4), as well as an indirect marker of cellular immunity, Interferon-gamma (IFN-γ), were examined using enzyme-linked immunosorbent assays (ELISAs) (Figure 8B). Given IL-4’s role in humoral immunity through Th2 cells and IFN-γ’s association with cellular immunity via Th1 cells, this experiment aimed to determine the predominant type of immunity activated [15,16]. It is well documented that elevated cellular immunity is crucial for antitumor efficacy [17,18,19].

Following vaccination, all cancer vaccine formulations led to an increase in immunoglobulins. Notably, the DMKD-PS formulation enhanced the levels of IgG2a and IgG2b, which are critical for antitumor immunity, signaling a strong secondary immune response. IgG antibodies play an essential role in secondary immunity, facilitating the rapid elimination of previously encountered antigens [20,21]. In contrast, IgM antibodies are primarily produced during the primary immune response and serve as the first line of defense, later switching to IgG (particularly IgG2a and IgG2b) to strengthen the immune response [22,23].

Despite these significant changes in immunoglobulin levels, there was no notable increase in IL-4 and IFN-γ levels. This could be due to the body’s homeostatic mechanisms, which tightly regulate cytokine secretion to prevent immune overstimulation that could lead to tissue damage or autoimmunity [24,25]. The lack of a substantial rise in IFN-γ levels may reflect a nuanced immune modulation, indicating a targeted or regulated cellular response [26,27]. This suggests that the immune system is balancing tumor eradication with the preservation of healthy tissue, possibly through mechanisms that are not entirely dependent on IFN-γ.

**Figure 9:**
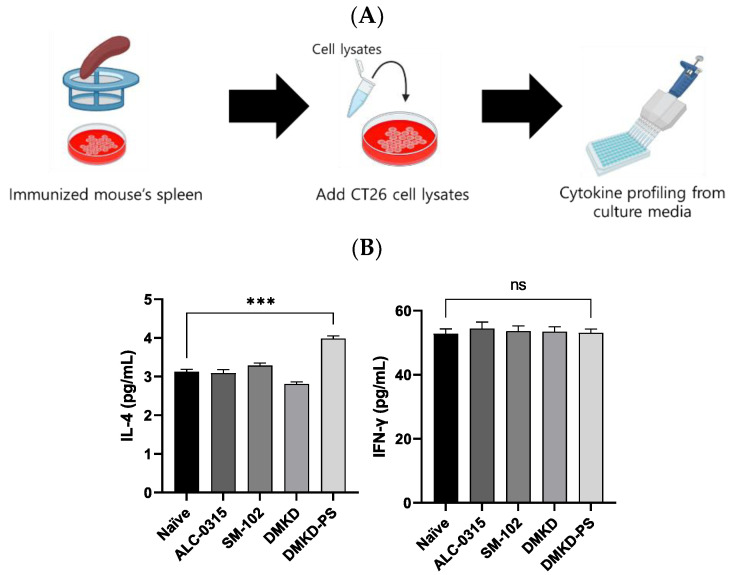
Cytokine profiling following MAGE-A3 mRNA anticancer vaccination from immunized splenocyte culture media. (**A**) A schematic representation of the culture process of the spleen derived from an immunized mouse and the subsequent cytokine profiling. (**B**) The cytokines IL-4 and IFN-γ in immunized splenocyte culture media were quantified. *** *p* < 0.001, ns—not specified.

Additionally, to verify CT26 tumor-specific immune responses induced by the mRNA vaccines, spleen cells were collected from vaccinated mice, cultured, and exposed to CT26 cell lysates to measure changes in IL-4 and IFN-γ (Figure 9A). The CT26 cell lysate, which contains the MAGE-A3 antigen, induced immune responses from the splenic immune cells. Enhanced cytokine expression in this context may serve as a strong indicator of selective immune activity against CT26 tumors. In the DMKD-PS group, IL-4 levels significantly increased, indicating a stronger humoral response, while IFN-γ levels exhibited an insignificant rise (Figure 9B). This pattern suggests a controlled immune response, potentially optimizing tumor targeting while minimizing collateral damage. It implies that the primary mechanism may involve multiple aspects of the immune system, including but not limited to IFN-γ.

## 3. Materials and Methods

### 3.1. Materials

1,2-Distearoyl-sn-glycero-3-phosphoethanolamine-N-[methoxy(polyethylene glycol)-2000] (DSPE-PEG_2000_-amine), cholesterol, 1,2-distearoyl-sn-glycero-3-phosphocholine (DSPC), and 1,2-dimyristoyl-rac-glycero-3-ethoxypolyethylene glycol-2000 (PEG-DMG) were purchased from Avanti Polar Lipid, Inc. (Alabaster, AL, USA). O,O’-dimyristyl-N-lysyl aspartate (DMKD) cationic lipid was provided by Prof. Yong Serk Park at Biomedical Laboratory Science, Yonsei University MIRAE Campus (Wonju, Republic of Korea). 2-Hexyl-decanoic acid, 1,1’-[(4-hydroxy butyl)imino]di-6,1-hexanediyl] ester (ALC-0315), 8-[(2-hydroxyethyl)[6-oxo-6-(undecyloxy)hexyl]amino]-octanoic acid, 1-octylnonyl ester (SM-102), and α-[2-(ditetradecylamino)-2-oxoethyl]-ω-methoxy-poly(oxy-1,2-ethanediyl) (ALC-0159) were purchased from Cayman Chemical (Ann Arbor, MI, USA). Cellomax^TM^ for the cell cytotoxicity test was supplied by Precaregene (Uiwang, Republic of Korea). Restriction enzymes (EcoRI, EcoRV, NotI, and SpeI), competent cells (*E. coli* DH5α), and EZ™ T7 High Yield In Vitro Transcription Kit were supplied by Enzynomics (Daejeon, Republic of Korea). pIVTRup was a gift from Ángel Raya (Addgene plasmid #101362; https://www.addgene.org/101362/#how-to-cite (accessed on 1 October 2024), RRID:Addgene101362). For the mRNA capping process, Ambion™ Cap Analog [m7G(5′)ppp(5′)G] was purchased from Thermo Fisher Scientific (Waltham, MA, USA). Dokdo Plasmid Mini-Prep Kits were purchased from ELPIS Biotech (Daejeon, Republic of Korea). The QiagenRNeasy Mini Kit (Hilden, Germany) and Toyobo 2× Thunderbird SYBR Green Master Mix (Osaka, Japan) were used for RT-qPCR. The ELISA kits for the analysis of IL-4 and IFN-γ were procured from Abcam (Cambridge, UK), whereas the IgG subtyping kit was purchased from Thermo Fisher Scientific.

### 3.2. Cell Lines and Cell Culture

Mouse macrophages (RAW 264.7, MAGE-A3-negative, and ATCC TIB-71^TM^) and mouse colorectal cancer cells (CT26, MAGE-A3-positive, and ATCC CRL-2638^TM^) were purchased from the American Type Culture Collection (ATCC; Manassas, VA, USA). RAW 264.7 and CT26 cells were cultured in Dulbecco’s modified Eagle’s Medium (BYLABS, Hanam-si, Republic of Korea) supplemented with 10% EqualFETAL^®^ bovine serum (Atlas biologicals, Fort Collins, CO, USA), and 100× Antibiotic–Antimycotic was diluted to 1× (Gibco™; Thermo Fisher Scientific). All cell lines were incubated at 37 °C in a humidified 5% CO_2_ incubator. The cells were subcultured to 80–90% confluency.

### 3.3. Animals

To prepare a tumor-syngeneic mouse model, 6-week-old male BALB/c mice were purchased from NARA Biotech (Seoul, Republic of Korea). All animal experiments were approved by the Institutional Animal Care and Use Committee (IACUC) of the Yonsei University MIRAE Campus (YWCI-202307-012-02) and performed in accordance with the guidelines and regulations.

### 3.4. Preparation of DMKD-PS

A mixture of DMKD, DSPE-PEG_2000_-amine, cholesterol, and PS (in mol %) was dried under a stream of nitrogen gas, followed by vacuum evaporation for 1 h. The dried lipid film was then rehydrated with 1 mL of normal saline solution. To form DMKD-PS liposomes, the suspension was subjected to sonication in a glass tube using a bath-type sonicator (Elma Schmidbauer GmbH, Singen, Germany) at 37 kHz and 60 W for three cycles of 15 minutes, with 10-minute intervals between each cycle. The resulting lipid suspension was then passed through a polycarbonate membrane with a pore size of 100 nm using an extruder (Avanti Polar Lipids, Alabaster, AL, USA) ten times. In addition, the other LNPs were prepared using alternative ionizable cationic lipids, such as ALC-0315 and SM-102, using the same protocol. Additionally, DMKD LNPs without PS were also produced. 

### 3.5. Synthesis of Melanoma-Associated Antigen A3 (MAGE-A3) mRNA

The MAGE-A3 sequence was cloned into the pIVTRup plasmid using a series of molecular biological steps. MAGE-A3 was amplified from CT26 cells by reverse transcription polymerase chain reaction (RT-PCR). The amplified product was then extracted from a 1% agarose gel to isolate specific DNA fragments. The isolated MAGE-A3 DNA fragment was inserted into the pIVTRup plasmid, which was previously digested with *Eco*RV. To confirm the successful insertion of MAGE-A3 into the plasmid, the final product was analyzed using 1% agarose gel electrophoresis. In addition, DNA sequencing was performed to ascertain the sequence of the inserted MAGE-A3. The T7 RNA polymerase IVT Kit was used for the in vitro translation of MAGE-A3 according to the protocol suggested by the manufacturer, and capping was performed at the 5′ end using the m7G capping reagent. Transcribed MAGE-A3 mRNA was purified by ethanol precipitation.

### 3.6. Western Blotting Analysis

The RAW 264.7 and CT26 cells were lysed using radioimmunoprecipitation assay (RIPA) buffer (iNtRON Biotechnology, Seongnam, Republic of Korea) with a 100× protease inhibitor cocktail (Thermo Fisher Scientific). The cell lysates were harvested in 1.5 mL tubes and then centrifuged at 14,000× *g* for 30 min at 4 °C. The supernatants were transferred to new microcentrifuge tubes, and the total protein concentration was determined using a bicinchoninic acid (BCA) protein assay (Thermo Fisher Scientific). The mixtures of protein solutions and 5× reducing dye (BYLABS, Hanam, Republic of Korea) were heated at 95 °C for 10 min. The mixtures (30 μg protein) were run on 10% SDS-PAGE gel at 100 V for 1.5 h and then transferred onto nitrocellulose (NC) membranes (Pall Corporation, Pensacola, FL, USA) at 350 mA for 1.5 h. The NC membranes were blocked with 1×Tris-buffered saline containing 0.1% Tween 20 (TBST) and 3% (w/v) skim milk (Oxoid Ltd., Basingstoke, UK) for 1 h at room temperature with an agitator. After blocking, the membranes were incubated with a 1:1000 dilution of anti-MAGE-A3 antibodies (Proteintech, Rosemont, IL, USA) and then with a 1:3000 dilution of anti-GAPDH mouse antibodies (Millipore, Darmstadt, Germany) in TBST containing 3% skim milk at 4 °C overnight. The membranes were washed three times with 1× TBST and then treated with a 1:5000 dilution of horseradish peroxidase (HRP)-conjugated anti-mouse antibodies (Cell Signaling Technology, Danvers, MA, USA) in 1× TBST containing 5% (w/v) skim milk for 1 h at room temperature. After treatment with the secondary antibody, the membranes were washed three times with 1 × TBST. Finally, the protein bands were visualized by SuperSignal^TM^ West Pico PLUS (Thermo Fisher Scientific) and detected using Fusion Solo Chemi-doc (Vilber Lourmat, Marne-la-Vallée, France). All the whole western blot figures can be found in the Supplementary Materials.

### 3.7. Quantitative Reverse Transcription Polymer Chain Reaction

Total RNA was isolated using the RNeasy Mini Kit (Qiagen) according to the manufacturer‘s guidelines. For cDNA synthesis, Reverse transcription master mix (ELPIS biotech, Daejeon, Republic of Korea) was used, and cDNA was produced using 1 μg of RNA. The primers used in this experiment for analyzing glyceraldehyde-3-phosphate dehydrogenase (GAPDH) and melanoma-associated antigen A3 (MAGE-A3) are detailed as follows. For GAPDH, the primer sequences were 5′-CGGGAA GCT TGT CAT CAA TGG-3′ (forward) and 5′-GGC AGT GAT GGC ATGG GAC TG-3′ (reverse). The primer sequences for MAGE-A3 were 5′-GAG TAC CTT GTT CCA ATC CCA G-3′ (forward) and 5′-CCA GGGG CAG TGA CAA GCA-3′ (reverse). All primers were purchased from Genotech (Daejeon, Republic of Korea). The reaction mixture for PCR included 10 μL of 2× Thunderbird SYBR qPCR Mix (Toyobo, Osaka, Japan), 2 μL of each primer (10 pmol/μL), 7 μL of distilled water, and 1 μL of template cDNA. The PCR reaction conditions were set to 95 °C for 3 min, and then 40 cycles of 95 °C for 3 s and 63 °C for 30 s, using the CFX96 Real-Time PCR System Detector (Bio-rad, Hercules, CA, USA). The RT-qPCR data were analyzed using the comparative Delta Ct method (2^−ΔCt^) with GAPDH as an endogenous control.

### 3.8. Animal Studies

An in vivo tumor model of male BALB/c mice was established for preventive and therapeutic cancer studies. For cancer prevention tests, the mRNA anticancer (5 μg of mRNA) vaccine was prepared at a concentration of 20 μg of total lipid in a 50 μL volume and injected into the thighs of the mice twice a week. Following the second vaccine administration, 2 × 10^5^ CT26 cells were subcutaneously inoculated, and subsequent changes in tumor size and weight were monitored for four weeks, with 12 measurements taken over the course. The immunized mice were injected with 5 × 10^4^ CT26 cells via the tail vein to establish a tumor metastasis model. For therapeutic tumor experiments, CT26 cells were transplanted subcutaneously into BALB/c mice. Just as the average tumor size exceeded 100 mm^3^, mRNA cancer vaccines were administered twice a week, and changes in tumor size and weight were tracked for up to four weeks. Notably, no additional chemotherapy was administered during this period, and the mice received only vaccine treatment.

### 3.9. Histological Analysis

Major organs and tumors were preserved by immersion in 4% formalin for 72 h, followed by a rinse with distilled water. The tissues were then dehydrated using a gradient of ethanol concentrations starting at 70% and ending at 100%. After dehydration, tissues were embedded in paraffin and sectioned using a microtome. The sectioned tissue slices were deparaffinized and stained with H&E reagents. Finally, the stained tissue sections were examined under a light microscope. Tumor tissues were embedded in paraffin blocks and sectioned for IHC analysis. The process began with antigen retrieval and blocking to prepare tissue sections. Subsequently, the sections were incubated with an anti-mouse MAGE-A3 antibody overnight at 4 °C. Subsequently, tissue sections were treated with HRP-conjugated anti-rabbit antibodies. Finally, sections were stained with DAB (3,3’-Diaminobenzidine).

### 3.10. Enzyme-Linked Immunosorbent Assay

A week after administering the cell lysate (equivalent to 2 × 10^5^ cells) into an immunized mouse, immunoglobulin subtyping was conducted using the immunized mouse serum via ELISA. Concurrently, the serum levels of IFN-γ and IL-4 were quantified in the same manner. To investigate the immune response of CT26 cells, primary culture was initiated following the extraction of the spleen from immunized mice. The cell lysate, equivalent to 2 × 10^5^ cells, was added and incubated for five days. IL-4 and IFN-γ levels were quantified using ELISA on the culture medium.

### 3.11. Statistical Analysis

Statistical analyses were performed using GraphPad Prism software version 9.3 (GraphPad Software, Boston, MA, USA). *Student t-deviation* (Student’s *t*-test) in a two-tailed manner was used for comparisons between the control and experimental groups. In this study, changes in tumor size in the animal experiments were analyzed using two-way ANOVA. The survival rate of the vaccinated mice was evaluated using the log-rank (Mantel–Cox) test, whereas cytokine levels determined by ELISA were analyzed using the paired *t*-test. *p*-values less than 0.05 were significant.

## 4. Conclusions

This study verified the prophylactic and therapeutic potentials of mRNA cancer vaccines formulated using lipid nanoparticles [14]. Compared to commercially available lipid nanoparticles such as ALC-0315 and SM-102, the novel formulation of phosphatidylserine-containing DMKD lipid nanoparticles could not only stably encapsulate mRNA molecules under refrigeration for several months but also more effectively maintain their transfection capability. Moreover, pre-inoculated MAGA-A3 mRNA molecules carried by most of the prepared lipid nanoparticles exhibited significant preventive effects on CT26 tumor progression for 28 days compared to the control. Moreover, mRNA treatment did not cause serious damage to the major internal organs. Notably, vaccination with DMKD-PS lipid nanoparticles appeared to be the most effective against tumor progression, including the metastasis of CT26 cells to the lungs. The anticancer preventive and therapeutic efficacy of MAGE-A3 mRNA in DMKD-PS lipid nanoparticles is presumably due to elevated humoral and cellular immune responses, such as increases in immunoglobulins IgG_2a_ and IgG_2b_ as well as changes in IL-4. In conclusion, DMKD-PS lipid nanoparticles could deliver MAGE-A3 mRNA molecules that were effectively expressed as tumor-associated antigens. This demonstrated that DMKD-PS lipid nanoparticles can be utilized as an efficient mRNA delivery modality for anticancer immunotherapy.

## 5. Patents

Park, Y. S., Choi, K. C., and Lee, D. H. (2022). Cationic lipid nanoparticles were used as mRNA vaccines (Korean Patent No. 10-2022-0044633). Korean Intellectual Property Office [28].

## Data Availability

Data are contained within the article.

## Supplementary Materials

The following supporting information can be downloaded at: https://www.mdpi.com/article/10.3390/cancers16193428/s1, Figure S1. All the whole western blot figures.

## References

[1] Sung H., Ferlay J., Siegel R.L., Laversanne M., Soerjomataram I., Jemal A., Bray F. (2021). Global Cancer Statistics 2020: GLOBOCAN Estimates of Incidence and Mortality Worldwide for 36 Cancers in 185 Countries. CA Cancer J. Clin..

[2] Schirrmacher V. (2019). From chemotherapy to biological therapy: A review of novel concepts to reduce the side effects of systemic cancer treatment (Review). Int. J. Oncol..

[3] Wyld L., Audisio R.A., Poston G.J. (2015). The evolution of cancer surgery and future perspectives. Nat. Rev. Clin. Oncol..

[4] Tohme S., Simmons R.L., Tsung A. (2017). Surgery for Cancer: A Trigger for Metastases. Cancer Res..

[5] Nurgali K., Jagoe R.T., Abalo R. (2018). Editorial: Adverse Effects of Cancer Chemotherapy: Anything New to Improve Tolerance and Reduce Sequelae?. Front. Pharmacol..

[6] Esfahani K., Roudaia L., Buhlaiga N., Del Rincon S.V., Papneja N., Miller W.H. (2020). A review of cancer immunotherapy: From the past, to the present, to the future. Curr. Oncol..

[7] Akkın S., Varan G., Bilensoy E. (2021). A Review on Cancer Immunotherapy and Applications of Nanotechnology to Chemoimmunotherapy of Different Cancers. Molecules.

[8] Saxena M., van der Burg S.H., Melief C.J.M., Bhardwaj N. (2021). Therapeutic cancer vaccines. Nat. Rev. Cancer.

[9] Liu J., Fu M., Wang M., Wan D., Wei Y., Wei X. (2022). Cancer vaccines as promising immuno-therapeutics: Platforms and current progress. J. Hematol. Oncol..

[10] Vishweshwaraiah Y.L., Dokholyan N.V. (2022). mRNA vaccines for cancer immunotherapy. Front. Immunol..

[11] Haen S.P., Löffler M.W., Rammensee H.-G., Brossart P. (2020). Towards new horizons: Characterization, classification and implications of the tumour antigenic repertoire. Nat. Rev. Clin. Oncol..

[12] Zajac P., Schultz-Thater E., Tornillo L., Sadowski C., Trella E., Mengus C., Iezzi G., Spagnoli G.C. (2017). MAGE-A Antigens and Cancer Immunotherapy. Front. Med..

[13] Conley A.P., Wang W.L., Livingston J.A., Ravi V., Tsai J.W., Ali A., Ingram D.R., Lowery C.D., Roland C.L., Somaiah N. (2019). MAGE-A3 is a Clinically Relevant Target in Undifferentiated Pleomorphic Sarcoma/Myxofibrosarcoma. Cancers.

[14] Choi K.C., Lee D.H., Lee J.W., Lee J.S., Lee Y.K., Choi M.J., Jeong H.Y., Kim M.W., Lee C.G., Park Y.S. (2024). Novel Lipid Nanoparticles Stable and Efficient for mRNA Transfection to Antigen-Presenting Cells. Int. J. Mol. Sci..

[15] Anovazzi G., Medeiros M.C., Pigossi S.C., Finoti L.S., Moreira T.M.S., Mayer M.P.A., Zanelli C.F., Valentini S.R., Rossa-Junior C. (2017). Functionality and opposite roles of two interleukin 4 haplotypes in immune cells. Genes Immun..

[16] Desmedt M., Rottiers P., Dooms H., Fiers W., Grooten J. (1998). Macrophages Induce Cellular Immunity by Activating Th1 Cell Responses and Suppressing Th2 Cell Responses1. J. Immunol..

[17] Koyama-Nasu R., Wang Y., Hasegawa I., Endo Y., Nakayama T., Kimura M.Y. (2022). The cellular and molecular basis of CD69 function in anti-tumor immunity. Int. Immunol..

[18] Lu C., Liu Y., Ali N.M., Zhang B., Cui X. (2023). The role of innate immune cells in the tumor microenvironment and research progress in anti-tumor therapy. Front. Immunol..

[19] Wang C.Q., Lim P.Y., Tan A.H.-M. (2024). Gamma/delta T cells as cellular vehicles for anti-tumor immunity. Front. Immunol..

[20] Leusen J.H.W., Nimmerjahn F., Nimmerjahn F. (2013). The Role of IgG in Immune Responses. Molecular and Cellular Mechanisms of Antibody Activity.

[21] Moreland L.W., Secondary immune response (2004). Rheumatology and Immunology Therapy.

[22] Liu J., Wang Y., Xiong E., Hong R., Lu Q., Ohno H., Wang J.Y. (2019). Role of the IgM Fc Receptor in Immunity and Tolerance. Front. Immunol..

[23] Keyt B.A., Baliga R., Sinclair A.M., Carroll S.F., Peterson M.S. (2020). Structure, Function, and Therapeutic Use of IgM Antibodies. Antibodies.

[24] Zaidi M., Yuen T., Sun L., Rosen C.J. (2018). Regulation of Skeletal Homeostasis. Endocr. Rev..

[25] Xiang Y., Zhang M., Jiang D., Su Q., Shi J. (2023). The role of inflammation in autoimmune disease: A therapeutic target. Front. Immunol..

[26] Jorgovanovic D., Song M., Wang L., Zhang Y. (2020). Roles of IFN-γ in tumor progression and regression: A review. Biomark. Res..

[27] Schroder K., Hertzog P.J., Ravasi T., Hume D.A. (2003). Interferon-γ: An overview of signals, mechanisms and functions. J. Leukoc. Biol..

[28] Park Y.S., Choi K.C., Lee D.H. (2022). Cationic lipid nanoparticles were used as mRNA vaccines. Patent.

