# Supplementary figures and images for: Innovative Cancer Immunotherapy with MAGE-A3 mRNA Cancer Vaccines
Source: Cancers (Basel). 2024 Oct 9;16(19):3428. doi: 10.3390/cancers16193428 (PMC11475142; doi:10.3390/cancers16193428)

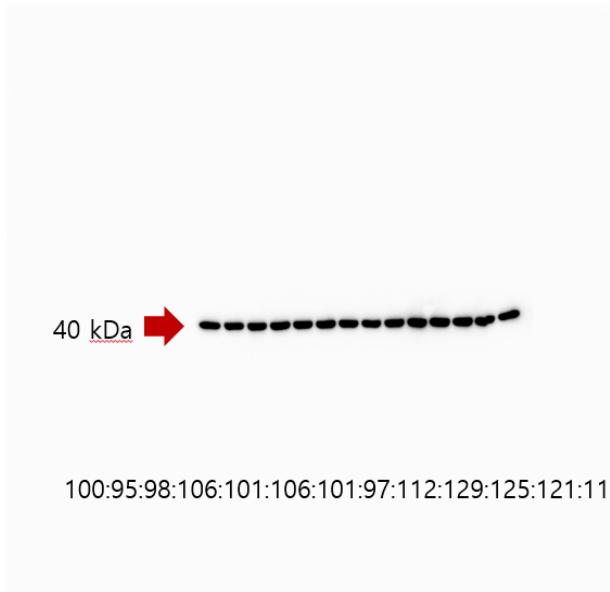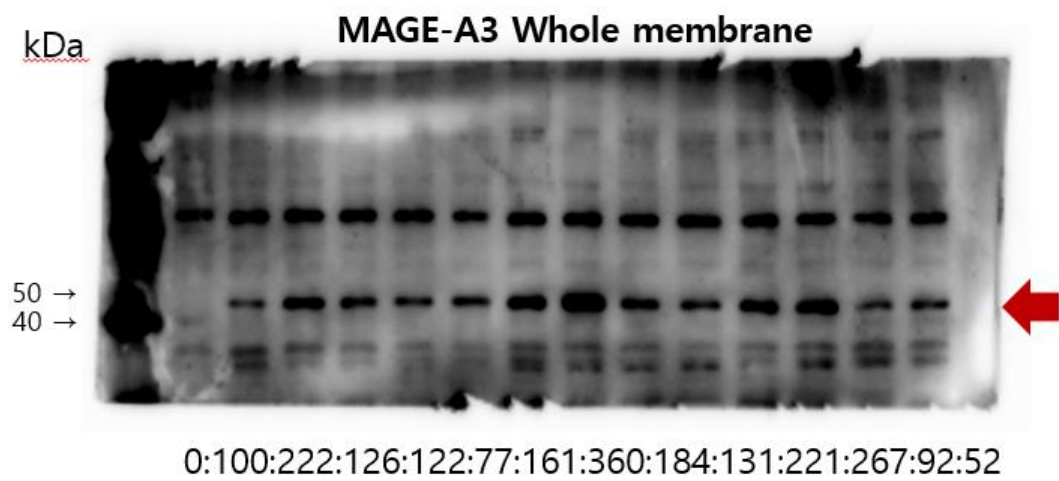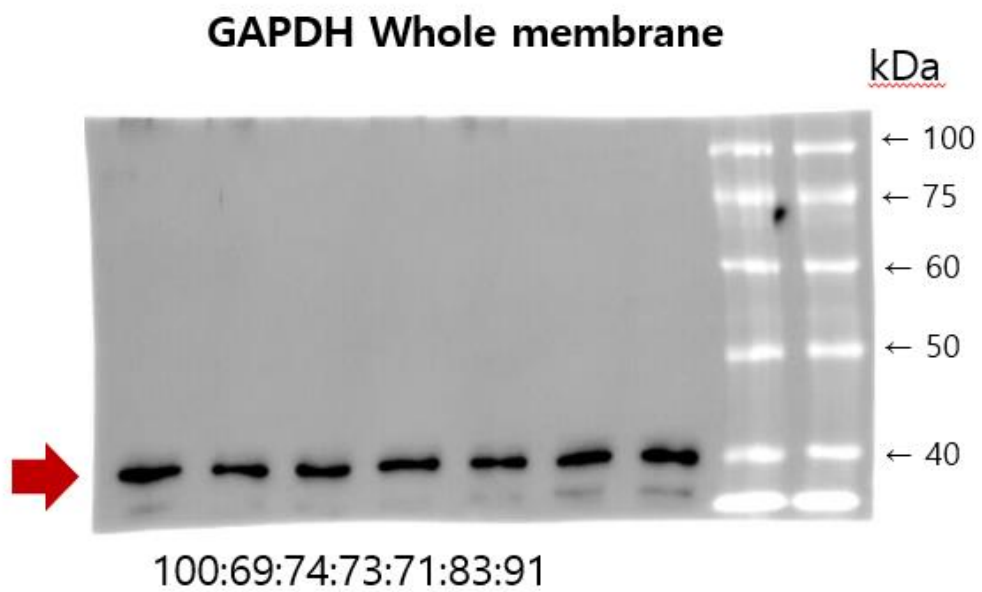

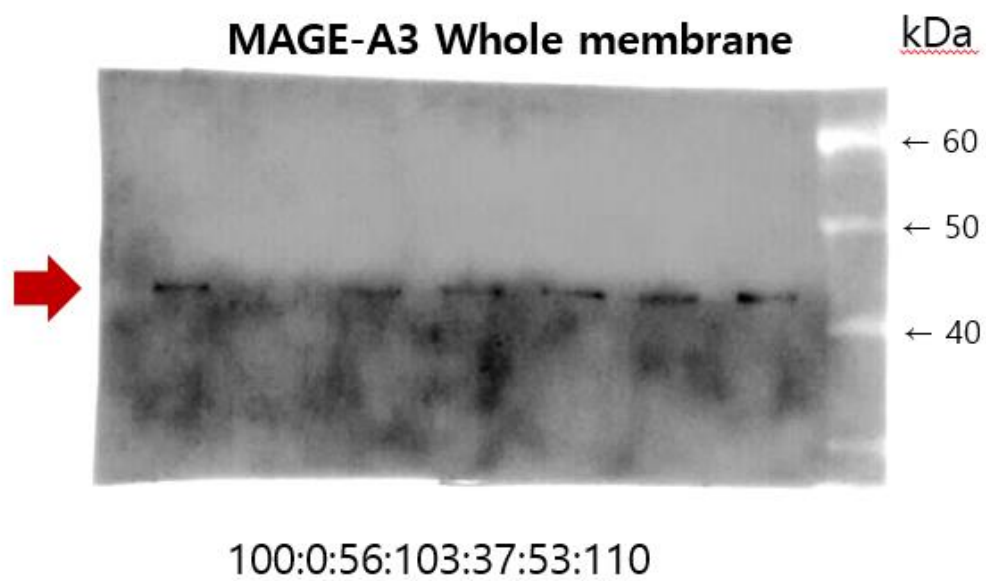

**Figure S1.** All the whole western blot figures.

Supplement: Supplementary file 1 [file cancers-16-03428-s001.zip › cancers-3244385-supplementary materials.pdf]
